# PEPFAR’s response to the convergence of the HIV and COVID‐19 pandemics in Sub‐Saharan Africa

**DOI:** 10.1002/jia2.25587

**Published:** 2020-08-07

**Authors:** Rachel Golin, Catherine Godfrey, Jacqueline Firth, Lana Lee, Thomas Minior, B Ryan Phelps, Elliot G Raizes, Julie A Ake, George K Siberry

**Affiliations:** ^1^ Office of HIV/AIDS United States Agency for International Development Washington DC USA; ^2^ Office of the U.S. Global AIDS Coordinator and Health Diplomacy Washington DC USA; ^3^ US Centers for Disease Control and Prevention Atlanta GA USA; ^4^ U.S. Military HIV Research Program Walter Reed Army Institute of Research Silver Spring MD USA

**Keywords:** COVID‐19, SARS‐CoV‐2, HIV care continuum, PEPFAR, readiness, response

## Abstract

**Introduction:**

The COVID‐19 pandemic reached the African continent in less than three months from when the first cases were reported from mainland China. As COVID‐19 preparedness and response plans were rapidly instituted across sub‐Saharan Africa, many governments and donor organizations braced themselves for the unknown impact the COVID‐19 pandemic would have in under‐resourced settings with high burdens of PLHIV. The potential negative impact of COVID‐19 in these countries is uncertain, but is estimated to contribute both directly and indirectly to the morbidity and mortality of PLHIV, requiring countries to leverage existing HIV care systems to propel COVID‐19 responses, while safeguarding PLHIV and HIV programme gains. In anticipation of COVID‐19‐related disruptions, PEPFAR promptly established guidance to rapidly adapt HIV programmes to maintain essential HIV services while protecting recipients of care and staff from COVID‐19. This commentary reviews PEPFAR’s COVID‐19 technical guidance and provides country‐specific examples of programme adaptions in sub‐Saharan Africa.

**Discussion:**

The COVID‐19 pandemic may pose significant risks to the continuity of HIV services, especially in countries with high HIV prevalence and weak and over‐burdened health systems. Although there is currently limited understanding of how COVID‐19 affects PLHIV, it is imperative that public health systems and academic centres monitor the impact of COVID‐19 on PLHIV. The general principles of the HIV programme adaptation guidance from PEPFAR prioritize protecting the gains in the HIV response while minimizing in‐person home and facility visits and other direct contact when COVID‐19 control measures are in effect. PEPFAR‐supported clinical, laboratory, supply chain, community and data reporting systems can play an important role in mitigating the impact of COVID‐19 in sub‐Saharan Africa.

**Conclusions:**

As community transmission of COVID‐19 continues and the number of country cases rise, fragile health systems may be strained. Utilizing the adaptive, data‐driven programme approaches in facilities and communities established and supported by PEPFAR provides the opportunity to strengthen the COVID‐19 response while protecting the immense gains spanning HIV prevention, testing and treatment reached thus far.

## 1 INTRODUCTION

Less than three months from the first reported cases of COVID‐19 in China, the pandemic reached Nigeria, the first sub‐Saharan African country to report a confirmed case of COVID‐19, at the end of February 2020 [1]. COVID‐19 response plans were rapidly developed and instituted as governments and donor organizations braced themselves for the impact of COVID‐19 in low‐ and middle‐income countries with high burdens of people living with HIV (PLHIV) [2, 3].

Despite sweeping measures to mitigate COVID‐19 transmission, as of 1 July 2020, 303,986 confirmed cases, and 6,155 deaths were reported across the World Health Organization’s African Region Member States [4], including 11 countries with a ≥5% adult HIV prevalence [5]. Information about the impact of HIV co‐infection on COVID‐19 transmission, morbidity and mortality is limited. By 20 April 2020, there were only eight case reports of COVID‐19/HIV co‐infection published in the scientific literature, none from Africa [6, 7, 8, 9]. As of June 2020, South Africa reported 2352 cases of COVID‐19 in PLHIV in the Western Cape, accounting for 18% of all COVID‐19 cases there [10].

Wider spread of COVID‐19 in Africa may result in millions of PLHIV being exposed to, and potentially infected with, SARS‐CoV‐2. There is uncertainty about its impact on this population and health systems directed to them. WHO recently projected up to 3.6–5.5 million hospitalizations and 190,000 deaths in Africa from COVID‐19 over the course of one year, if control measures are inadequate [11].The potential negative impact of COVID‐19 in these countries is currently uncertain, but COVID‐19 is anticipated to contribute both directly and indirectly to the morbidity and mortality of PLHIV, requiring countries to leverage existing resources and infrastructure to propel COVID‐19 responses while safeguarding PLHIV and HIV programme gains.

## 2 DISCUSSION

### 2.1 Potential and known adverse impact of COVID‐19 on HIV programmes and services

Although it remains unclear whether PLHIV have increased risk of SARS‐CoV‐2 acquisition or progression to severe disease [12], emerging evidence from South Africa suggests there may be a modest increase in mortality associated with HIV, irrespective of ART use or viral load suppression especially in the presence of other comorbidities [10]. It is clear, however, that PLHIV are at high risk of suffering the consequences of COVID‐19’s detrimental impact on weak, overburdened health systems. While most (approximately 80%) patients with COVID‐19 have mild illness, others require hospitalization for supplemental oxygen and critical care interventions [13]. This estimate of distribution of COVID‐19 severity is based largely on the experience in Asia, Europe, and North America. Health system demands may be quite different in African countries based on demographics (generally younger populations) and burden of communicable and non‐communicable diseases. Hospital beds in the sub‐Saharan countries supported by the United States President’s Emergency Plan for AIDS Relief (PEPFAR) range from 0.3 beds per 1000 people in Ethiopia to 2.8 beds per 1000 people in South Africa [14]. The health care workforce is similarly limited. In Malawi there are 0.02 physicians and 0.25 nurses/midwives per 1000 people [15, 16]. Health systems strengthening efforts of large, successful public health programmes such as PEPFAR have been predominantly focused on outpatients, and significant gaps in critical care capacity persist in low‐and‐middle income countries [17].

Of further concern, laboratory instruments, supplies and staff needed for HIV viral load (VL) monitoring, early infant HIV diagnosis (EID), and tuberculosis testing may be diverted to SARS‐CoV‐2 testing. The net result will be a dangerous inability to meet the prevention, testing and treatment needs for either HIV or COVID‐19. In the West Africa experience with Ebola, modelling suggests more deaths were attributed to disruptions in HIV, TB, and malaria services than directly to Ebola infection [18].

As in the West African Ebola outbreak [18], fears of healthcare‐associated transmission may exacerbate healthcare worker shortages and discourage people from seeking crucial HIV services at facilities. Additionally, healthcare workers have been disproportionately impacted by COVID‐19 due to insufficient quantities of personal protective equipment and resulting exposure [19]. COVID‐19 mitigation strategies are limiting importation and distribution of critical health commodities required for essential HIV services [20]. Furthermore, concerns have been raised that COVID‐19 plans will divert resources from HIV, TB and malaria programmes [21].

### 2.2 Rapid adaptation of PEPFAR guidance during COVID‐19

In anticipation of these disruptions, PEPFAR established guidance to rapidly adapt HIV programmes to maintain essential HIV services while protecting recipients of care and staff from COVID‐19. The general principles prioritize protecting recent gains in the HIV response while minimizing exposure to COVID‐19 at healthcare facilities and reducing the burden on these facilities [22, 23, 24]. Congruent to recent advocacy by the International AIDS Society [25], there is special emphasis on continuity of treatment for PLHIV through expanded use of decentralized and multi‐month dispensing (MMD) of ART. Uninterrupted delivery of essential prevention services, including infant HIV prophylaxis, HIV pre‐exposure prophylaxis, ante‐ and postnatal care, family planning and childhood immunizations is also prioritized, with clear guidance to decentralize these services to the greatest extent possible. Recommendations for virtual clinical monitoring and other essential services, including laboratory support and commodities management, have been included.

Psychosocial support services delivered through virtual platforms such as phone calls and social media platforms (e.g. Facebook and WhatsApp) are replacing in‐person services while reducing the risk of SARS‐CoV‐2 transmission to clients and staff. COVID‐19 control strategies have resulted in reported increases in domestic violence [26] and can increase child protection risks [27]. Orphans, vulnerable children, adolescent girls and young women (AGYW) have been prioritized to receive virtual risk screening and linkage to essential services, including referrals if at risk for or experiencing abuse, neglect or violence. Specific guidance has been provided on how to deliver virtual support to beneficiaries of PEPFAR’s Determined, Resilient, Empowered, Mentored and Safe (DREAMS) programme for AGYW [28].

### 2.3 Leveraging existing HIV platforms to help the COVID‐19 response while protecting essential HIV gains

To help mitigate the adverse impact of COVID‐19 on HIV services, programmes can leverage existing PEPFAR‐supported HIV platforms to accelerate COVID‐19 mitigation and response plans while concurrently protecting essential HIV gains. Specific platforms include the PEPFAR‐ supported laboratories, supply chain management systems, strategic information platforms, clinical service delivery and health worker investments, community outreach platforms and other health systems that allow for early HIV detection, surveillance, disease classification and treatment optimization. This comprehensive approach promotes country ownership, local leadership and stakeholder coordination for effective and sustainable responses. Many fundamental principles of the HIV response also apply to the COVID‐19 response: test symptomatic or at‐risk individuals, utilize contact tracing to reach others potentially affected, and offer an immediate intervention package (Table 1).

**Table 1:** Parallels between HIV epidemic control and approaches to containing COVID‐19

Principle	HIV epidemic control	COVID‐19 analogue
Identify symptomatic and at‐risk individuals	Provider‐initiated testing & counselling Voluntary counselling & testing	Testing of healthcare workers, or people presenting to points of entry Testing of symptomatic patients
Contain spread through contact tracing methods	Testing of biological children and sexual contacts of index cases (“Index Testing”) Contact tracing for PLHIV co‐infected with TB	Contact tracing and testing of known contacts
Rapid provision of a package of interventions	Linkage facilitation and same day and rapid ART initiation SMS adherence support Treatment of comorbidities and opportunistic infections for those with advanced disease	Self‐isolation for asymptomatic positives and contacts with SMS‐based check‐ins for clinical deterioration Supplementary oxygen, ventilatory support and/or investigational therapy (where appropriate)

ART, antiretroviral therapy; COVID‐19, coronavirus disease 2019; HIV, human immunodeficiency virus; PLHIV, people living with HIV; SMS, short message service; TB, tuberculosis.

PEPFAR‐funded partners have demonstrated responsiveness and agility and the below discussion includes information from PEPFAR programmes and partners about strategies they have undertaken to adapt HIV programming in response to COVID‐19.

### 2.4 Clinical, laboratory and supply chain platforms

PEPFAR continues to support host countries to implement innovations to meet the needs of PLHIV and those at risk of HIV infection. Differentiated service delivery for PLHIV through such interventions considers the individual’s specific needs and supports HIV case identification, treatment retention and viral suppression. Although PEPFAR is funding measures such as mobile phone appointment scheduling applications to promote social distancing within facilities, local restrictions can preclude individuals from seeking HIV testing and treatment at hospitals and clinics.

In response to reduced access to facility‐based services, strategic adaptions to testing services include leveraging private public partnerships and community platforms for distribution of HIV self‐testing kits in accordance with national guidance. Decentralized distribution of HIV self‐testing kits has provided a valuable platform to broaden COVID‐19 health messaging, screening and contact tracing. HIV self‐testing is also being leveraged to assist with index testing, especially in settings where active or assisted index texting is not feasible or safe. COVID‐19 programme adaptions have also required the decentralization of ART initiation sites, which has promoted targeted community‐based ART initiation initiatives by PEPFAR‐supported implementing partners in the Democratic Republic of the Congo, Eswatini, Malawi and South Africa.

Expanded treatment services include MMD of medications and distribution of medications through local pharmacies, community groups and community pharmaceutical lockers. To decongest facilities, limit COVID‐19 transmission and increase resources for COVID‐19 screening and treatment, many countries’ mitigation and response plans have accelerated MMD implementation. A minimum of 19 PEPFAR sub‐Saharan host countries have implemented, reinforced or increased three‐ to six‐month dispensing eligibility over recent months, with many adopting policy changes during COVID‐19 programmatic adaptions (Burundi, Democratic Republic of the Congo, Eswatini, Ethiopia, Ghana, Kenya, Lesotho, Liberia, Malawi, Mali, Mozambique, Namibia, Rwanda, South Africa, South Sudan, Tanzania, Togo, Zambia and Zimbabwe).

While some phlebotomy services have been reduced due to COVID‐19‐related service disruptions and limited protective personal equipment, PEPFAR continues to promote the continuity of essential viral load monitoring through decentralized specimen collection points and telephonic follow‐up once results are received. Several PEPFAR implementers across sub‐Saharan Africa have made a concerted effort to align viral load specimen collection with a client’s multi‐month ART refill date. These interventions help PLHIV avoid the need to frequently queue at clinics and allow facilities to focus more on COVID‐19 screening and treatment.

PEPFAR‐supported implementing partners continue to work with national and local governments to strengthen service delivery and develop a strong integrated clinical‐laboratory interface that facilitates patient care (e.g. VL and tuberculosis testing) and case surveillance. Diagnosis of active SARS‐CoV‐2 infection is performed through polymerase chain reaction technology; PEPFAR partners are adapting the same infrastructure and laboratory systems supported by PEPFAR for HIV EID, VL and tuberculosis testing, when appropriate, to rapidly implement and scale capacity for SARS‐CoV‐2 diagnostic testing. One example of adapted clinical‐laboratory collaboration is in Burundi, where PEPFAR is supporting near real time facility‐level laboratory commodities monitoring and modifying both client flow and sample transport to accommodate social distancing measures.

Additionally, many PEPFAR partners assist national HIV programmes with robust procurements coupled with monitoring and reporting systems to ensure delivery and distribution of essential laboratory commodities and ARVs. These same systems can be utilized and modified to help mitigate stockouts of critical medical supplies during the COVID‐19 response. Specific modifications currently being implemented include utilizing mobile message platforms (e.g. SMS and WhatsApp) to monitor stock levels and providing increased technical assistance to support distribution of commodities.

### 2.5 Community platforms

HIV programmes have long focused on understanding the epidemic and which populations are at risk. These programmes identify and engage vulnerable populations and offer education about risk and prevention measures as well as HIV testing and treatment services. Through longstanding engagement with governments, local health systems, NGOs, civil society organizations and advocacy groups, PEPFAR and its implementing partners have built trust with communities, including marginalized members such as key populations, and orphans and vulnerable children, resulting in a unique, sustained platform for public health messaging and demand creation.

In response to COVID‐19, these community partners have made a deliberate effort to capacitate community health workers in PEPFAR host countries. Community lay cadres in Ethiopia, for example, are learning how to protect themselves and the households they serve through training sessions on COVID‐19 risk factors, signs and symptoms and mitigation strategies. PEPFAR implementing partners continue to promote hygiene through the provision of cloth face masks and hand sanitizer for those working in communities.

The existing systems to create demand and capacity for HIV pre‐exposure prophylaxis, voluntary male medical circumcision, testing and rapid linkage to treatment are being utilized to spread messaging to populations about COVID‐19 prevention, testing and medical care; and in a complementary manner, COVID‐19 messaging campaigns have been identified by PEPFAR partners to be a valuable opportunity to reinforce the need for HIV prevention, testing and treatment services, especially for harder to reach sub‐populations, such as men. Across many sub‐Saharan countries, PEPFAR‐funded mobile phone platforms are helping sustain necessary services by enabling bidirectional community and facility linkages and delivering mHealth messages. The virtual community reach is widened in parts of Eswatini, Lesotho and Zambia through radio communication campaigns that provide information on COVID‐19 risk and prevention information and when to seek medical care; and radio messages in Tanzania and Uganda reinforce the need for and availability of essential HIV services.

### 2.6 Food and nutritional security platforms

In response to ongoing concern over the unknown economic impact of COVID‐19 among vulnerable individuals, including those living with HIV, PEPFAR established guidance for implementing partners to integrate food security and nutritional assessments into routine in‐person and virtual follow‐up. PEPFAR‐supported organizations continue to provide approved food and nutritional activities in close coordination with broader US Government‐funded food assistance programmes. PEPFAR is also considering additional funding requests to help mitigate HIV treatment interruption due to food insecurity, especially in light of importing, exporting and distribution challenges experienced at major sub‐Saharan African ports and transport hubs.

### 2.7 Strategic information platforms

PEPFAR’s data‐driven programming closely monitors key indicators and outcomes, including HIV diagnoses, linkage to ART and virological suppression. The institutionalized data systems and culture of granular analysis informs evidence‐based implementation. Even in the midst of COVID‐19 disruptions, PEPFAR‐supported implementing partners continue to routinely analyse their own programme data, often in concert with local health officials, and adapt their HIV services accordingly. These best practices and PEPFAR’s commitment to data‐driven public health action is being leveraged by emergency operation centres and “situation rooms” to monitor the spread and impact of COVID‐19 disease. Continued triangulation and analysis of HIV and COVID‐19 data is required as countries promptly respond to both pandemics. Program adaptations include utilization of mobile phone applications for data entry, decentralized data reporting centres, and videoconference platforms to analyse data and refine programming accordingly.

### 2.8 Human resources for health platforms

Aligned with host countries’ needs, PEPFAR’s current human resources for health (HRH) strategy includes HRH capacity assessment and strengthening, improving HRH information systems and data utilization, investing in HRH retention, service quality improvements, and sustainable financing for healthcare workers who deliver HIV services [29]. PEPFAR has supported nearly 300,000 healthcare workers at more than 3000 laboratories and 70,000 health facilities [30]. PEPFAR‐supported health workers, laboratory personnel, supply chain advisors and policy makers have equipped countries to be able to respond to HIV and emerging infectious diseases such as COVID‐19. The COVID‐19 pandemic has required mobility and flexibility in service delivery teams, with task sharing and redeployment in response to illness and social disruption. These health professionals have nimbly adapted to the changed environment through use of healthcare workforce tools to adjust staffing needs and monitoring resulting staffing adjustments to provide uninterrupted delivery of priority HIV services [31].

## 3 CONCLUSIONS

There remain many unknowns during the rapidly evolving COVID‐19 pandemic, especially in sub‐Saharan Africa where high rates of COVID‐19 among PLHIV are expected. HIV service delivery adaptions resulting from COVID‐19 disruptions require close monitoring and evaluation. PEPFAR‐supported platforms are mitigating COVID‐19’s adverse impacts on HIV services as well as on PLHIV themselves by building upon 17 years of cross‐cutting lessons learned and best practices. As COVID‐19 spreads among vulnerable populations and strains fragile health systems, PEPFAR’s responsive, data‐driven facility and community programming is being leveraged to protect existing HIV investments through the provision of uninterrupted essential HIV service delivery, while also significantly strengthening host countries’ COVID‐19 responses.

## COMPETING INTERESTS

The authors declare no competing interests.

## AUTHORS’ CONTRIBUTIONS

The initial concept for this commentary was conceived by GKS. RG, CG, TM, BRP and GKS contributed to the initial outline. All authors (RG, CG, JF, LL, TM, BRP, EGR, JAA, GKS) contributed to the initial manuscript content. RG, CG, BRP, ER and GKS contributed to the revisions. All authors (RG, CG, JF, LL, TM, BRP, EGR, JAA, GKS) approved the final commentary.
